# Demonstrating library value: the development of a customizable Library Value Planner

**DOI:** 10.29173/jchla29825

**Published:** 2025-08-01

**Authors:** Mark Mueller, Nicole Askin, Jeanna Hough, Brooke Ballantyne Scott, Joan Bartlett

**Affiliations:** 1 Saskatchewan Health Authority, Health Sciences Library, Regina, SK; 2 Winnipeg Regional Health Authority Virtual Library, Winnipeg, MB; 3 Halton Healthcare Clinical Library, Halton, ON; 4 Fraser Health Authority, New Westminster, BC; 5 McGill School of Information Studies, Montreal, QC

## Abstract

**Background:**

Library professionals in the health sciences sector need to strategically plan and map out library services. Each library and their parent organization have unique needs and service offerings.

**Objective:**

To develop an adaptable Library Value Planner (LVP) tool based on the Levels of Library Service benchmarking document developed by the Health Science Information Consortium (HSIC) that can be used for (i) strategic and operational planning and (ii) mapping out needs for implementing new library services in individual contexts.

**Methods:**

This project involved: (i) searching the literature; (ii) analyzing current trends and best practices in Canadian health libraries; (iii) updating and renaming of the Levels of Library Service document; (iv) drafting and disseminating a French and English survey; (v) leading French and English focus groups; (vi) analyzing the feedback received from the surveys and focus groups, and (vii) revising the tool based on this feedback.

**Results:**

The results from the surveys and the focus groups showed that participants were satisfied with the versatile nature of the LVP. Some respondents expressed concerns about the formatting of the LVP and others were not sure how and when the LVP ought to be used. This feedback highlighted the need to develop and disseminate education for library professionals about the tool.

**Conclusion:**

The CHLA/ABSC Standards Standing Committee developed a flexible and robust tool that, when paired with education, can be used to advocate and demonstrate the value of library services in the health sciences.

## Introduction

Library professionals have an ongoing need to strategically plan and map out library services in the health sciences. Each library and its parent organization is unique and will have different needs and service offerings [1]. The Canadian Health Libraries Association Standing Standards Committee (CHLA/ABSC SSC) developed the Library Value Planner (LVP) tool, based on the Health Science Information Consortium’s (HSIC) Levels of Library Service document, that can be used and adapted to each library’s individual context. The goal was to create a flexible and robust tool that, when paired with education, could be used to advocate and demonstrate the value of library services in the health sciences sector. Given recent closures of health sciences libraries [2], it is more important than ever to be able to demonstrate the value of the library to decision-makers.

### Background

The LVP was developed from previous work on Canadian health sciences library standards. The Canadian Health Libraries Association/Association des bibliothèques de la santé du Canada (CHLA/ABSC) started a Standards Task Force (ST) in 2019 with a mandate to review and update the 2006 CHLA-ABSC Standards for Library and Information Services in Canadian Healthcare Facilities. The ST disbanded after the updated Standards were released in 2020. The CHLA/ABSC created a new Standards Standing Committee (CHLA/ABSC SSC) that same year with mandates to offer practical application, updates, and support to the 2020 Standards and to ensure the currency of the Standards.

An additional mandate was to further the Association’s goal of demonstrating value and advocacy over the long term [3], including:

Develop practical tools to help libraries apply the Standards—with reference to the Health Science Information Consortium Library Value Toolkit, and ‘Levels of Library Service’ service example visualization. [4]

The Levels of Library Service (LLS) visualization tool was created by the Health Science Information Consortium (HSIC)^2^, an Ontario collective of information services in hospitals, public health units, and other healthcare institutions. In March 2013, the HSIC established a “Measuring Value Taskforce” (Taskforce)^3^, which defined a framework that libraries could use to conceptualize measuring their value.

**Fig. 1:**
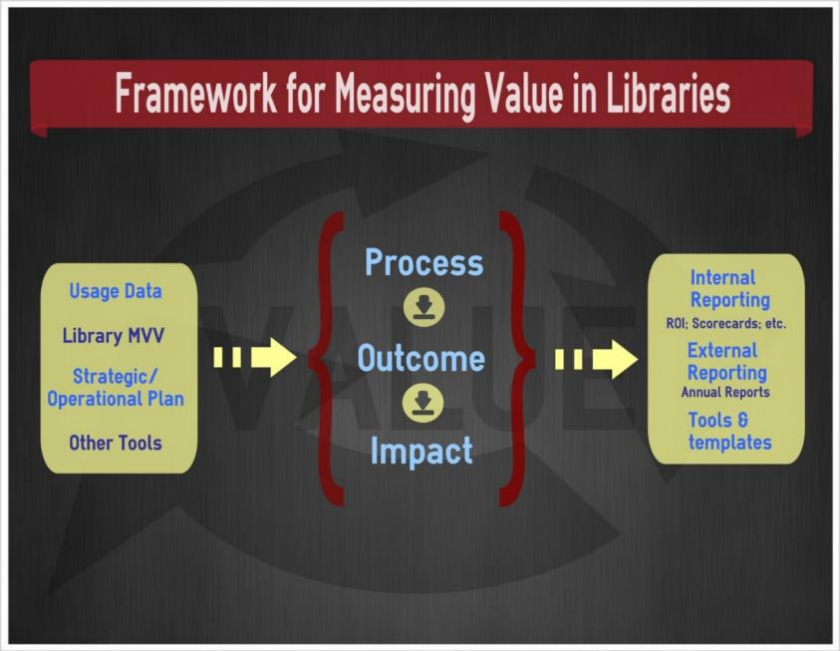
Framework for Measuring Value in Libraries, HSIC

The final framework (Figure 1) lists the inputs (resources provided to the library) in the left column, the outputs (the degree to which the library’s resources and services are utilized) in the right column and the process by which users can measure their value in the centre column [5].

Based on the concepts outlined by the framework, the Taskforce created a Library Value Toolkit that contained templates, practical examples, personal anecdotes from each of Taskforce’s members, and links to existing tools, calculators, recommended readings, and top tips. The guiding ideas around the Library Value Toolkit were presented at the 2014 CHLA/ABSC conference in Montreal:

- Not intended to be comprehensive; meant as a starter, practical toolkit
- Flexible for the end user
- Maintenance would involve updating links, adding new content (fully every two years)
- Submit a Resource: allowing site visitors to suggest useful content (reviewed semi-annually for appropriateness and inclusion on the site) [6]

**Fig. 2:**
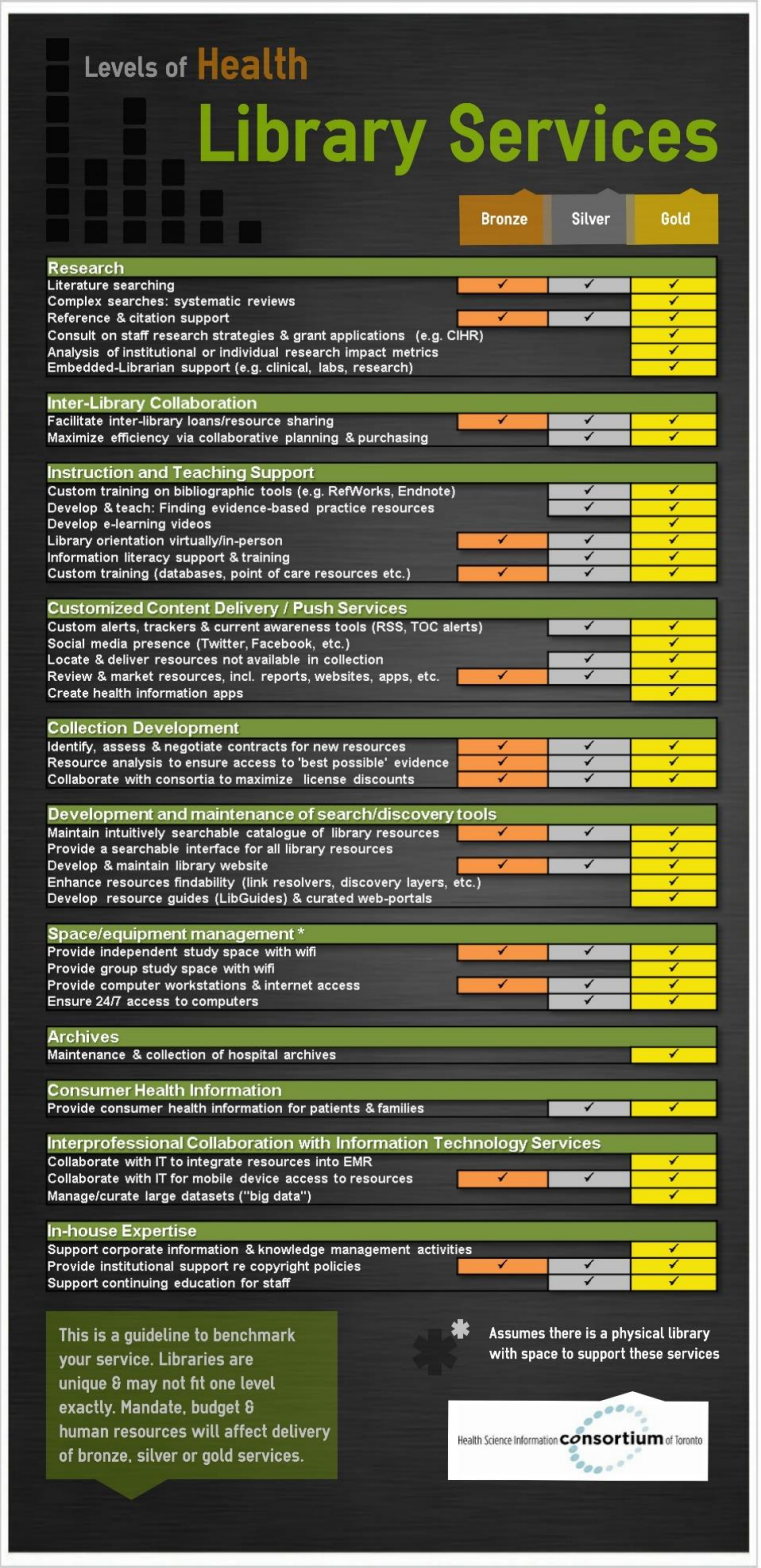
Levels of Library Service infographic

The Taskforce met at regular intervals to brainstorm examples to correspond with the shortlisted selection of resources. One such example was the Levels of Library Service infographic (Figure 2). This infographic was a proof-of-concept example of a type of visualization members could create for their own organizations, according to their own identified services, to present to their respective reporting structures.

The Library Value Toolkit was formally launched at the 2014 CHLA/ABSC conference and received very positive feedback at the event and over the ensuing years; anecdotally, some member librarians cited use of the Library Value Toolkit as an integral part of their work to establish continued funding and institutional support for library services. The Taskforce handed the Library Value Toolkit over to the HSIC Professional Practice Committee in 2015 when the Taskforce disbanded. Unfortunately, the scope of maintenance was not feasible for an already busy committee and the Toolkit became outdated. In 2020, with permission from HSIC, ownership of the Library Value Toolkit moved to CHLA/ABSC to maintain the toolkit’s currency and to support resources for the new Standards on a national level.

It became clear that there was a need to both update the HSIC Toolkit and expand the Levels of Library Service from a simple infographic to a practical and customizable planning tool. Given the relevance to the CHLA/ABSC Standards, the SSC decided to focus on this expansion and developed the LVP from 2020 to 2023. The LVP took the form of a much more comprehensive spreadsheet that members could complete and customize according to their parent organizations and library services, using the LVP to present their current state, their value-add, or to develop business cases for additional services.

### Literature review

The authors conducted a literature review to contextualize the LVP with other value measurement tools published within the last 20 years. A literature search was performed in February 2024 in the following databases: CINAHL (EBSCO); Library and Information Science Abstracts (LISA, EBSCO); Library, Information Science and Technology Abstracts (LISTA, EBSCO); and MEDLINE (OVID). Google, Google Scholar, the ISO Standards, and Internet Archive were also searched. The authors searched the following broad concepts using a combination of keywords and controlled subject headings when applicable: (i) health sciences libraries and librarians; (ii) value, output, and performance; and (iii) measurement tools. While preference was given to resources pertaining to the field of health sciences librarianship, relevant resources produced by and for other library sectors, such as the ISO Standards, were not excluded.

Three systematic reviews were identified that provided an outline of the following five measures for determining the strategic value and performance of libraries: input, output, process, outcome, and impact [1,7,8]. Input, output, and process outline how well a library performs as an institution. Input measures reflect the type of resources provided to the library (i.e. budget, staff, collections, facilities, technology, etc.) [1]. While input measures are useful in measuring library assets, they do not provide any indication as to how these resources are being used or how effective they are in meeting user needs [9]. Output measures reflect the degree to which a library’s resources and services are utilized [1]. These measures consider the number of items loaned, reference interactions, instructional sessions, gate counts, computer logins, website visits, and similar statistics [9].

Some libraries may rely on process (or efficiency) measures to calculate the time or cost per activity [1,7]. An example of a process measure is the return-on-investment calculators developed by Library Research Service and the National Library of Medicine [10,11], which are used to calculate the efficiency and effectiveness of a library’s budget policies [11]. Other examples of process measures include benchmarking studies that assess how libraries perform according to standards set by professional organizations [12] and how well they compare with other libraries [8,13,14]. These types of measures are useful to help libraries determine best practices in the profession, make management and staffing decisions, conduct research projects, and determine the current status of the library [8,12-14]. While these process measures are useful to library managers in making decisions for their staff and users, they can be difficult to define and present in a way that is meaningful to institutional managers [1]. Additionally, benchmarking studies may not take into consideration the unique needs and demands of specific libraries [14]. Comparative data is also problematic because it is difficult to maintain momentum in collecting statistics and convince organizational leadership of the importance of contributing to benchmarking [12,13]. Hospital libraries must articulate and demonstrate the value of their services in alignment with the organizational strategic objectives, outcomes, and value for money by stating why the library exists and not simply what they do [15].

The other two measures identified from the systematic reviews that determine the library’s impact on the individual level and organizational level may provide insights as to “why the library exists” [16]. Libraries need to define outcome measures that are relevant to their institution and assess the extent to which those outcomes are met [16]. Outcome measures may be useful to institutional managers as they reflect the degree to which the library’s services impact their users on the individual level (i.e. attitudes, knowledge, professional skills, values, etc.) [1,17]. Some of these measures may involve the use of the Critical Incident Technique (CIT) where respondents are asked to think about a specific instance where the library’s resources and services impacted them most [1]. This technique has been validated in many studies [5] and is used in Canada, the U.S., and the U.K. [16–19]. One potential limitation of CIT is that asking respondents to think back on a specific event may lead to problems with memory bias or respondents potentially creating a positive description of certain events to please the researchers [18]. Impact measures consider how the library’s resources and services affect the broader community and organization over a longer period (i.e. learning, economic) or impact the broader community [1,9]. These types of measures are important because it is easier for stakeholders to see the relevance of and need for such measures and are therefore more likely to view the assessments in a positive way [15]. Dalton et al. developed a set of generic outcome-based performance measures for Irish hospital libraries that linked the impact of library services with measurable healthcare outcomes and objectives aligned with the mission and goals of the organization [16]. Lewis et al. used balanced scorecards in four North American universities that evaluated performance in four areas: finance, learning and growth, customers, and internal processes. These balanced scorecards provided indicators to enable these libraries to develop more structured planning processes aligned with organizational performance [10]. The National Health Service libraries in the U.K. developed a value and impact toolkit with guidance and tools for helping libraries measure value based on organizational outcomes (i.e. balanced scorecards, Critical Incident Technique, impact assessments, and more) [21]. The International Organization for Standardization outlined a wide range of methods to gather evidence about the outcomes and impacts of library collections, services, and spaces to help libraries with strategic planning, promoting library value to support stakeholder decisions on levels of service, and achieving strategic goals [15].

Matthews concludes that determining library value is a complicated process that depends on the perspective of the individual attempting to assign or quantify value, and that “any attempt to establish the value of library outcomes should recognize that a combination of perspectives–educational, informative, recreational, cultural, and public benefits–will result in a more realistic assessment of value” [1]. When assessing value, libraries need to first determine what they want to measure and demonstrate, who the value and impact stakeholder is, and what method and tool they want to use [20]. Strategic planning is also complex and involves careful assessment of service effectiveness and cost-benefit. In her study of academic medical libraries, Piorun found that staff used performance indicators tied to strategic objectives to assess progress on goals [21]. Harrison et al.’s assessment of health libraries in Ireland found that staff perceived that the value of health libraries was not well understood by policymakers; the authors concluded that “building of an evidence base of successful projects and the strategic dissemination of evidence” should be a key goal for their national library association [22].

## Methods

**Fig. 3:**
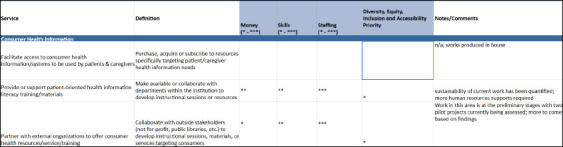
Example section from the LVP showing the resourcing allocated to consumer health information services at a theoretical library

The CHLA/ABSC SSC used the HSIC Levels of Library Service infographic as a starting point when developing the LVP. The infographic was reviewed line-by-line to identify services provided by health and social services libraries that were absent; these were then added into the expanded list. The definitions and wordings of the list items were clarified and reorganized into overarching modules during this iterative process. The LVP was reframed from evaluating the level of services provided by a library to outlining the resources (money, staff, and skills) that are or would be required to offer each service (Figure 3). Throughout this process, the SSC sought to emphasize the descriptive nature of the LVP and the flexibility of its application to various types of libraries.

The SSC developed README documentation and case studies to help users understand how to adapt the tool to their individual contexts. The LVP and the accompanying materials were translated into French and both English and French versions were posted onto the CHLA/ABSC SSC website, alongside other materials adapted from the former HSIC Library Value Toolkit.

In consultation with the CHLA Board, feedback on the LVP was sought from the broader CHLA/ABSC community. Cross-country online-facilitated focus groups were planned via Zoom in both French and English. Invitations were shared via CHLA/ABSC communication channels, particularly the member listserv. Target participants included Canadian library staff in any health library setting. An accompanying feedback survey (Online Supplement, Appendix 1) was sent for those unable to attend the focus groups. Facilitation questions and associated probes for the focus groups (Online Supplement, Appendix 2) were predetermined and two committee members served as facilitators for both French and English groups. The survey included a mix of qualitative and quantitative questions; all ten questions were optional.

Focus groups (one English, one French) were conducted using the CHLA Zoom account in July 2023; the meetings were recorded and automatically transcribed within Zoom. The survey was delivered via SurveyMonkey between July and September 2023, with invitations and reminders shared via the CHLA/ABSC listserv. Both focus group participants and the survey respondents consented to participate in the study. Approval was received for the study via the Research Ethics Board at McGill University and secondarily the University of Manitoba and Halton Healthcare.

Transcripts from the focus groups and survey responses were combined, summarized, and organized by one committee member, with qualitative responses grouped into positive and negative categories and then sorted into subgroups by theme. The qualitative summary was coded according to whether the concerns were best addressed by changes to the tool itself, changes to the associated documentation (case studies and README), or via user education.

## Results

**Fig. 4:**
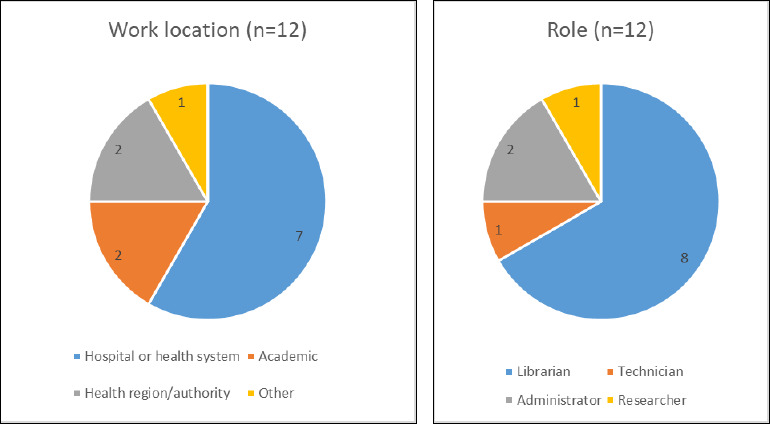
Graph of survey respondents

Two participants (one per language) completed the focus groups; one additional person consented to participate in the English group but withdrew midway through. Thirty respondents consented to participate in the survey; however, the post-consent questions received between 10 and 13 responses only. The demographics of the survey responses are summarized in Figure 4.

In response to “I understand how the tool can or should be used” (n=13), the majority of respondents (n=7) agreed or strongly agreed. For “The sections and services are understandable” (n=10), five agreed. For “The tool is user-friendly in its present form” (n=12), five agreed. For “The tool is practical in its present form” (n=12), six agreed. These responses are detailed in Table 1.

**Table 1:** Survey responses

	Strongly disagree	Disagree	Neutral	Agree	Stronglyagree
I understand how the tool can or should be used	1	4	1	6	1
The sections and services are understandable	0	3	2	6	0
The tool is user-friendly in its present form	1	4	2	5	0
The tool is practical in its present form	1	2	3	6	0

Eleven respondents weighed in on whether there were missing sections or services: ten respondents said there were no missing sections and eight no missing services. In the free-text comments for these questions only one respondent identified a service that they felt was missing (human resources management) and other comments seemed to contradict the “yes” response. For example, the one respondent who replied that there were missing sections said in their free-text response, “I don’t think there are missing sections, but I believe there may be too many subcategories”.

The qualitative survey responses were combined with the comments from the focus groups for the purposes of analysis. Positive commentary highlighted the perceived flexibility of the tool and its formatting. Many respondents felt the sections were clear and that having definitions alongside services improved comprehensibility of the LVP. Most of the respondents praised the exhaustive nature of the LVP: one respondent commented that it was “interesting to see the options that I had never even thought about”. Another said, “Once you understand how to use it, it is easy to use”, particularly given the examples provided by the case studies. Overall, respondents felt that it was helpful “to have all health libraries across Canada using the same tool to be able to understand what others are doing”. Respondents anticipated using the tool in a variety of ways, including advocating for more resources, communicating with leadership, benchmarking services, strategic planning, evaluating personnel skills and tasks, and getting ideas about additional services to add to their library’s current service offerings.

Negative commentary addressed themes such as the technical and formatting issues of the LVP and confusion regarding the LVP’s use and purpose; for example, one respondent suggested using the “freeze panes” feature in Excel to ensure top headers would remain visible when scrolling, while another asked, “What does it mean whether I have 1 star or 3?”. Respondents expressed concern communicating about the tool with non-library management staff and noted the use of jargon as a barrier to communication. Some were unsure what to enter in each column, or how to adapt the tool to their context.

The SSC sorted the negative feedback according to whether it could be addressed in the LVP itself, the accompanying documentation, or via user education. Formatting and wording concerns were addressed within the LVP, most significantly in the name of the tool. The original name of the LVP was “Library Value and Resource Validation Tool”; this was amended to the “Library Value Planner” as it was felt that this name would be more descriptive of the tool’s function. The language used in the LVP was reviewed to remove the “library jargon” and confusing language. Some of the formatting issues in the LVP were then addressed: freeze panes were added to the document and the French and English versions were separated into downloadable documents to address usability concerns. The tool was also reviewed and revised with specific regard to its applicability to non-academic libraries, as this was a concern raised by several respondents.

Similar to the LVP, there were also some formatting concerns with accompanying documentation (README–amended to “How the Planner Works”–and case studies), and the language was amended to remove “library jargon” and include plain-language explanations of what the LVP is and what it is intended for to improve comprehensibility. The case studies were revised to make them more targeted to help users understand how to apply the LVP to their own contexts and purposes.

The qualitative responses also underscored a significant need for dedicated user education. A synchronous workshop and accompanying resources were created to train users on how to understand the LVP and apply it to their individual contexts; this workshop has so far been offered twice via Zoom to CHLA/ABSC members in November 2024. The following themes from the negative responses are being addressed through these workshops: (i) clarifying the purpose of the tool; (ii) demonstrating how the tool can be used to communicate with non-library management; (iii) clarifying how the tool is used; (iv) connecting the tool to the CHLA/ABSC Standards; (v) demonstrating how to adapt the tool to a particular context; (vi) and explaining how to rate the level of resources required.

## Discussion

The goal of this project was to create a strategic planning tool–the Library Value Planner–that all staff at health sciences libraries can use to document their current services and their potential growth, and the staffing, skills, and budgetary requirements to achieve these goals. Different libraries will have different services depending on their available resources and the visions, missions, and mandates of their libraries and parent organizations. The term “value” is problematic because “value” will have a different connotation according to the context and who it is that is asking the question. Ayre et al. also noted that the library literature has been grappling with the concepts of library value and their measurement for the past 20 years [12]. Further it would be nearly impossible for the creators of the planner to create an exhaustive list of services in a one-size-fits-all benchmarking tool that can be applied to all health sciences libraries in Canada. The LVP provides a list of services that “can be offered”, not what “must” be offered. As a strategic planning tool, not a benchmarking tool, the LVP is meant to assist libraries in defining and planning services and programs relevant to their institutions for strategic purposes.

The LVP can be used as a launching pad to stimulate discussions and generate ideas to help libraries with their strategic planning, and can be used as a complement with other strategic planning tools (e.g., SWOT Analysis, Gantt Charts, SBARS) to help libraries understand their contexts, their users, and to prioritize services that are most important to them and their communities. Given ongoing cuts and closures to Canadian health libraries [2], this work is vital to ensure the sustainability and future of library services.

### Limitations

The major limitation of this project was the low participation rate for both the survey and the focus groups and therefore the responses collected may not be representative of the opinions of the community as a whole. Because demographic data other than library and position type was not collected, other positionalities cannot be assessed which may impact interpretation of the LVP; for example, it is not possible to assess the usability of the tool for users with a first language other than English or French. Similarly, although efforts were made to ensure the CHLA/ABSC SSC members represented different library types and geographic regions, their personal perspectives may have biased the development of the LVP. Further feedback from the community will be vital to assess whether the revised LVP meets the needs of its intended users; this project provides a starting point.

### Future Directions

The LVP is a tool to complement the CHLA Standards and other benchmarking resources for health sciences libraries. Future work will assess use of the tool in Canadian health science library contexts and feedback from users in practice. The CHLA/ABSC SSC will also monitor the emerging roles of library and information professionals; the integration of new and disruptive technologies into library services; the evolution of user needs and interests; and the evolution of the language and terminologies library professionals use to describe their roles. These new terminologies and language will be integrated into the LVP to ensure that the content is familiar and relevant to all users. This evolution may result in the development of new benchmarking tools and resources that libraries in Canada may use to measure and advocate for their library services. These evolutions and developments could present both opportunities and threats to health sciences libraries in Canada that may require libraries to respond and advocate for their services in new and creative ways. Finally, the CHLA/ABSC SSC will monitor the literature for any new benchmarking and measurement tools that libraries can use to demonstrate and advocate their value to their stakeholders. These resources will be added to the Library Value Toolkit section on the CHLA/ABSC SSC website. The SSC will also continue to develop and deliver workshops and training materials designed to teach users how to use the LVP for strategic planning purposes.

Developing the language to describe the various phenomena in the LVP was a difficult process and monitoring the literature to help develop language that is familiar to all users will also be useful to ensure that the LVP is relevant to all users. Further validation and feedback from the CHLA community is part of an ongoing process to establish a rigorous evidence base that will keep the LVP relevant and current.

### Conclusion

The LVP is a visual strategic planning tool that was developed by the CHLA/ABSC SSC. It is intended to be used as a complement to the CHLA Standards and other tools to help libraries identify the resources required for strategic and operational purposes. The LVP is a versatile tool that enables its users to adapt and edit for their own context; this is what sets the LVP apart from other tools. The information that users attain from this tool can be used for planning, quantifying services and resources, advocacy, and allocating or re-allocating library resources. By using the LVP in combination with the CHLA Standards and other tools, health sciences library professionals can paint an in-depth and accurate picture of their individual library’s contributions to their parent organizations, which is key to advocating for the future of health science libraries.

## Supplementary Material





## Data Availability

An anonymized version of the survey responses is available on OSF: https://osf.io/85ykn/.

## Footnotes

2 Previously called Health Science Information Consortium of Toronto (HSICT) but now known as the Health Science Information Consortium (HSIC); will be referred to as such throughout the remainder of this paper.
