# Supplementary material for: Demonstrating library value: the development of a customizable Library Value Planner
Source: J Can Health Libr Assoc. 2025 Aug 1;46(2):11–20. doi: 10.29173/jchla29825 (PMC12352445; doi:10.29173/jchla29825)
Supplement: Supplementary file 1 [file JCHLA-46-011-s001.pdf]

Appendix 1: Feedback survey

Refer to the LVRVT tool, found at https://standards.chla-absc.ca/levels-of-library-service/

Consultez l'outil OMVRB, disponible à l'adresse suivante : https://standards.chla-absc.ca/fr/levels-of-library-service/

Questions

1. I understand how the tool can or should be used/Je comprends comment l'outil peut ou doit être utilisé
   a. Strongly disagree/Pas du tout d'accord, Disagree/Pas d'accord, Neutral/Neutre, Agree/D'accord, Strongly agree/Tout à fait d'accord (multiple choice options)
   b. Comments/Commentaires (free text field)
2. The sections and services in the tool are understandable/Les sections et les services de l'outil sont compréhensibles
   a. Strongly disagree/Pas du tout d'accord, Disagree/Pas d'accord, Neutral/Neutre, Agree/D'accord, Strongly agree/Tout à fait d'accord (multiple choice options)
   b. Comments/Commentaires (free text field)
3. The tool is user-friendly in its present form/L'outil est convivial dans sa forme actuelle
   a. Strongly disagree/Pas du tout d'accord, Disagree/Pas d'accord, Neutral/Neutre, Agree/D'accord, Strongly agree/Tout à fait d'accord (multiple choice options)
   b. Comments/Commentaires (free text field)
4. The tool is practical in its present form/L'outil est pratique dans sa forme actuelle

a. Strongly disagree/Pas du tout d’accord, Disagree/Pas d’accord, Neutral/Neutre, Agree/D’accord, Strongly agree/Tout à fait d'accord (multiple choice options)

b. Comments/Commentaires (free text field)

5. Are there any missing sections in the tool at this point? Y a-t-il des sections manquantes dans l'outil à ce stade?

    a. No/Non (multiple choice options)

    b. Yes/Oui

        i. Please explain/Veuillez expliquer votre réponse (free text field if Yes is selected)

6. Are there any missing services at this point?

    a. No/Non (multiple choice options)

    b. Yes/Oui

        i. Please explain, including the section in which the missing services should appear/Veuillez expliquer votre réponse, y compris la section dans laquelle les services manquants doivent figurer (free text field if Yes is selected)

7. Are there any points needing clarification? Y a-t-il des points à clarifier?

    a) Comments/Commentaires (Free text field)

8. How do you anticipate using the tool? How will it impact your library? Comment pensez-vous utiliser l'outil? Quel sera l'impact sur votre bibliothèque? (free text)

9. What type of library do you work in or with? Dans quel type de bibliothèque travaillez-vous? (multiple choice)
    a. Hospital or hospital system/Hôpital ou système hospitalier
    b. Health region/authority/Région/autorité sanitaire
    c. Health sciences academic/Universitaire, sciences de la santé
    d. Public library/Bibliothèque publique
    e. Federal government/Gouvernement, fédéral
    f. Provincial government/Gouvernement, provincial
    g. Other/Autre (free text)
10. What is your role? Quel est votre rôle?
    a. Administrator/Administrateur.trice
    b. Librarian/Bibliothécaire
    c. Library technician or assistant/Technicien.ne ou assistant.e de bibliothèque
    d. Other/Autre (free text)
