# Supplementary material for: Demonstrating library value: the development of a customizable Library Value Planner
Source: J Can Health Libr Assoc. 2025 Aug 1;46(2):11–20. doi: 10.29173/jchla29825 (PMC12352445; doi:10.29173/jchla29825)
Supplement: Supplementary file 2 [file JCHLA-46-011-s002.pdf]

Appendix 2: Focus group questions

English

1. Intro: what types of libraries and roles are represented here today?
2. What are your general thoughts/impressions of the tool?
3. Do you feel the tool and its components are understandable? *Probe: options (skills, personnel, financial, DEIA)*
4. Is anything missing from the tool? Any sections missing? Any services missing? If so, from which section? Anything that should be removed (or redundant)?
5. What did you find most useful in the LVRVT?
6. What are your thoughts on the user-friendliness of the tool?
7. What are your thoughts on the practical application of the tool? Validity/sustainability/adaptability of the tool for your particular context?
8. How do you anticipate using the tool? What will its impact be on your library or practice? (Discuss if we have time)
9. Suggestions on how to keep the tool current? (Discuss if we have time. May come out of eight)

French

1. Introduction : quels types de bibliothèques et de rôles sont représentés ici aujourd'hui?

2. Quelles sont vos idées/impressions générales sur l'outil?
3. Pensez-vous que l'outil et ses composants sont compréhensibles? *Point de discussion : options (compétences, personnel, finances, ÉDIA)*
4. Y a-t-il des éléments manquants dans l'outil ? Y a-t-il des sections manquantes? Des services manquent-ils? Si oui, dans quelle(s) section(s)? Y a-t-il des éléments qui devraient être supprimés (ou qui sont redondants)?
5. Qu'avez-vous trouvé de plus utile dans le l'OMVRB?
6. Que pensez-vous de la convivialité de l'outil?
7. Que pensez-vous de l'application pratique de l'outil? Validité/durabilité/adaptabilité de l'outil à votre contexte particulier?
8. Comment pensez-vous utiliser l'outil? Quel sera son impact sur votre bibliothèque ou votre pratique? (À discuter si nous avons le temps)
9. Suggestions sur la manière de maintenir l'outil à jour? (À discuter si nous avons le temps. Peut venir de la huitième question)
